# Supplementary material for: Basal Cell-Contact Dynamics Influence Tissue Packing in a Proliferating Mammalian Epithelium
Source: bioRxiv. 2025 Jul 31:2025.07.30.665610. Preprint. [Version 1] doi: 10.1101/2025.07.30.665610 (PMC12324399; doi:10.1101/2025.07.30.665610)
Supplement: 1 [file NIHPP2025.07.30.665610v1-supplement-1.pdf]

bioRxiv preprint doi: https://doi.org/10.1101/2025.07.30.665610; this version posted July 31, 2025. The copyright holder for this preprint (which was not certified by peer review) is the author/funder, who has granted bioRxiv a license to display the preprint in perpetuity. It is made available under aCC-BY 4.0 International license.

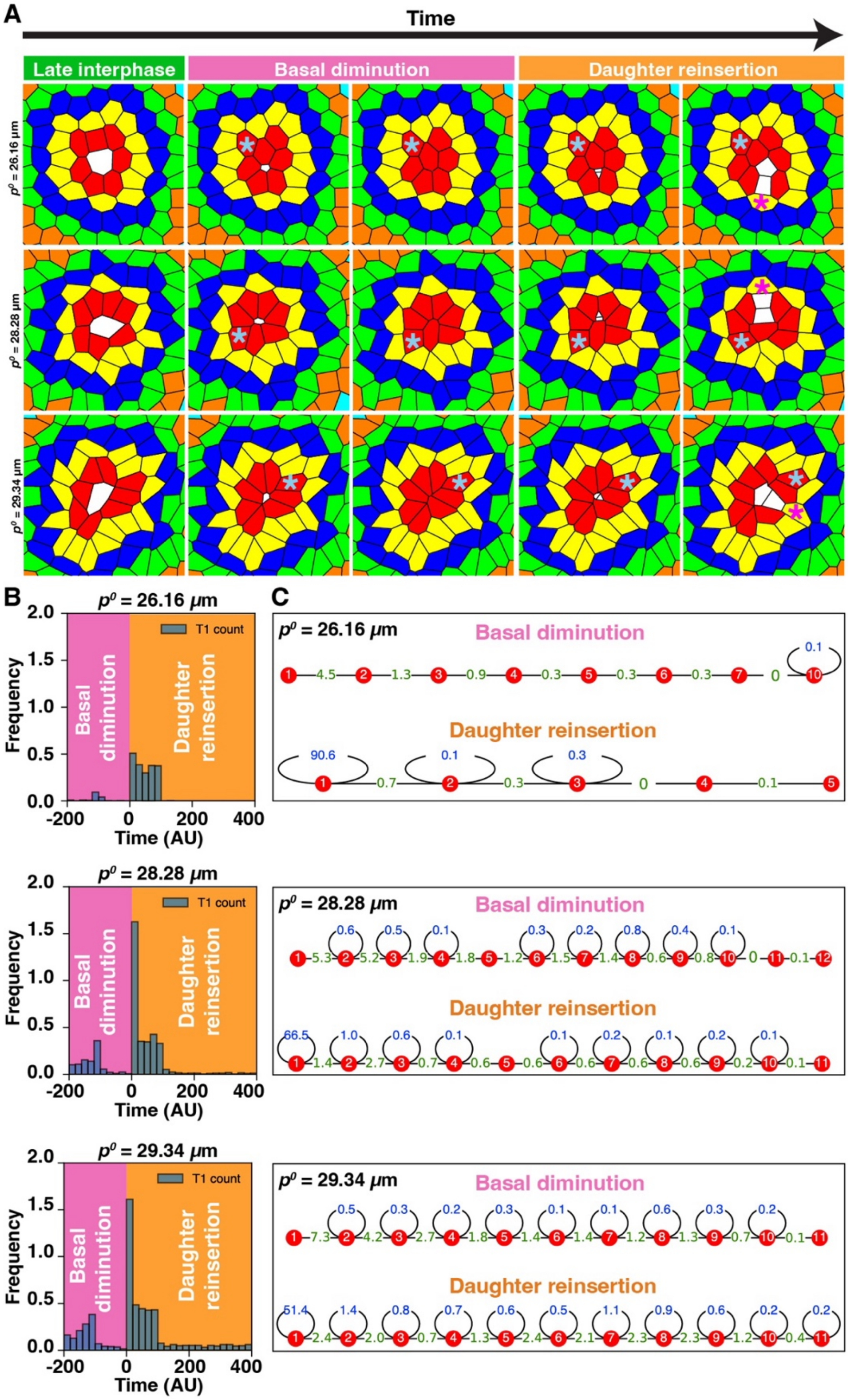

Figure S1. Vertex model simulations predict mitosis-induced basal cell-contact remodeling across a range of mechanical regimes.
(A) Snapshots of cell divisions in tissues with varying p_i^0 ($26.16\mu m, 28.28\mu m$, and 29.34μm). Mitotic cell: white; color-coded neighbors as in Fig. 1C. Asterisks mark T1 transitions.
(B) Temporal distribution of T1 transitions for each p_i^0. Negative and positive times indicate diminution and reinsertion, respectively (n = 324).
(C) Spatial distribution of T1 transitions as a function of topological distance from the mitotic cell.

bioRxiv preprint doi: https://doi.org/10.1101/2025.07.30.665610; this version posted July 31, 2025. The copyright holder for this preprint (which was not certified by peer review) is the author/funder, who has granted bioRxiv a license to display the preprint in perpetuity. It is made available under aCC-BY 4.0 International license.

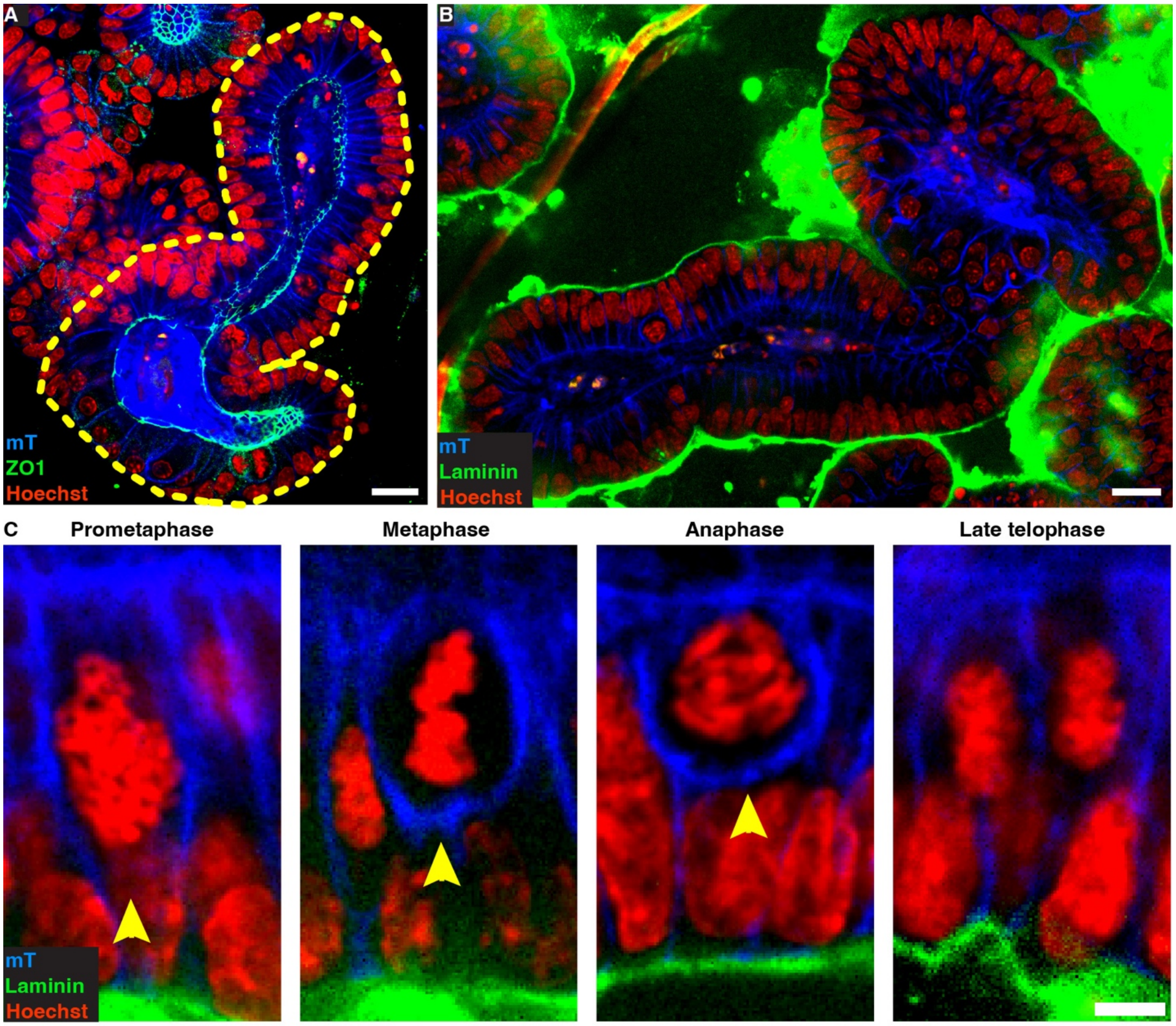

Figure S2. Proliferating epithelial cells from intestinal organoid culture are translocated from the basal region during the start of prophase and reinserted back into the basal region after cytokinesis.

(A, B) Intestinal organoid epithelium strained for ZO1 (n = 16 organoids) to identify the apical domain (A) and laminin (n = 14 organoids) to identify the basement membrane (B). The yellow outline marks the organoid boundary. All scale bars, 20 μm

(C) Mitotic cell body (arrowheads) is displaced away from the basement membrane at the start of mitosis. After cytokinesis, the daughter cell bodies return to the basal plane.

bioRxiv preprint doi: https://doi.org/10.1101/2025.07.30.665610; this version posted July 31, 2025. The copyright holder for this preprint (which was not certified by peer review) is the author/funder, who has granted bioRxiv a license to display the preprint in perpetuity. It is made available under aCC-BY 4.0 International license.

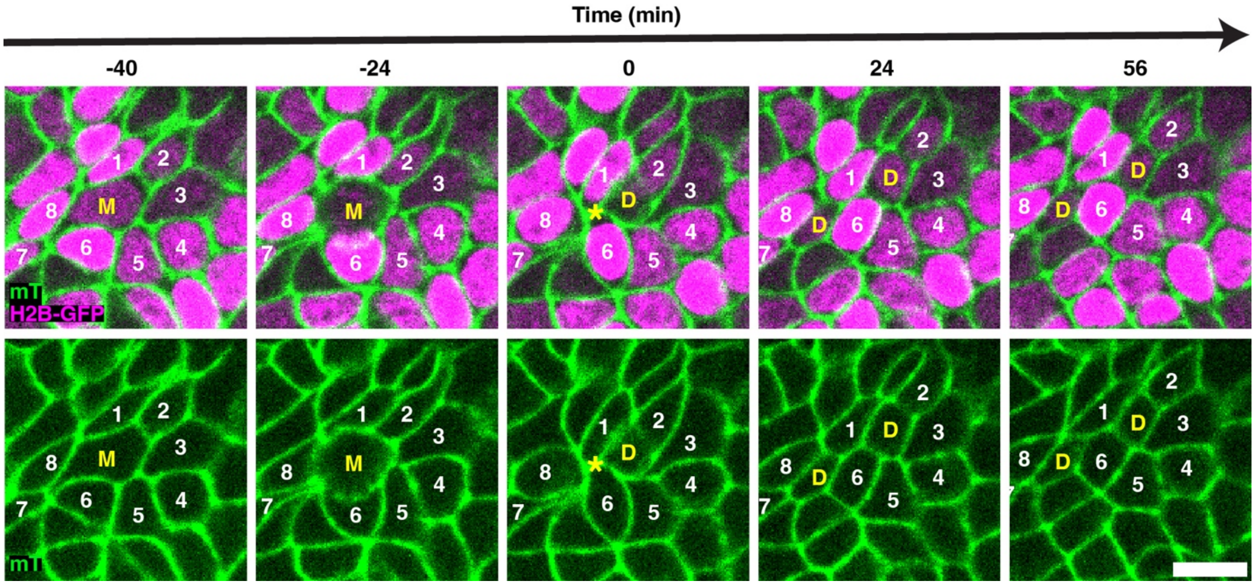

Figure S3. Daughter cell separation at the basal plane is an infrequent topological outcome. Timelapse images showing the basal cell-contact topology of a mitotic cell (‘**M’**), its immediate neighbors (‘**1-8’**), and daughter cells (‘**D’**). Membranes: mTomato (green); nuclei: H2B-GFP (magenta). Scale bar, 10 μm.

bioRxiv preprint doi: https://doi.org/10.1101/2025.07.30.665610; this version posted July 31, 2025. The copyright holder for this preprint (which was not certified by peer review) is the author/funder, who has granted bioRxiv a license to display the preprint in perpetuity. It is made available under aCC-BY 4.0 International license.

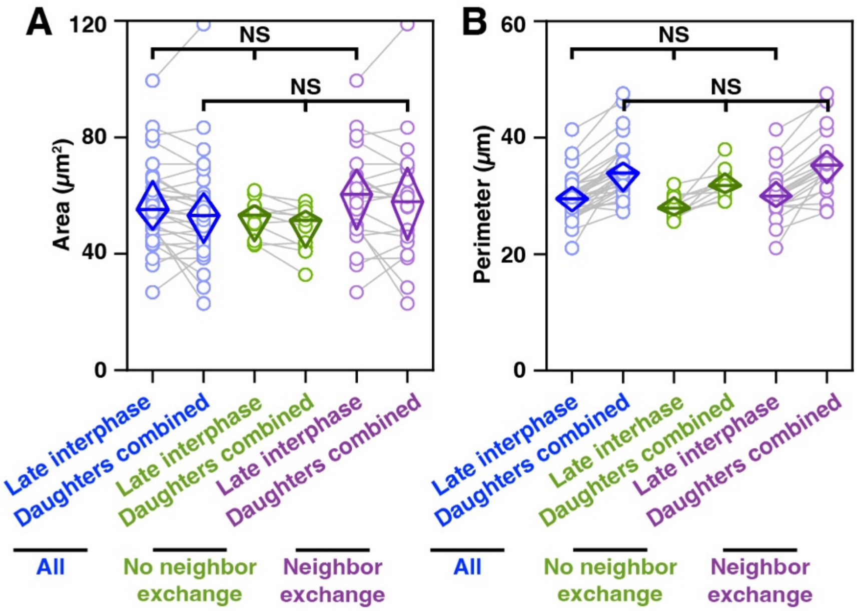

Figure S4. Basal surface area and perimeter of mitotic cells remain conserved across neighbor exchange conditions

Mother cell area (A) and perimeter (B) before mitosis compared to that of the daughter cells combined. The data is segregated based on whether (green) or not (magenta) neighbors exchanged after daughter cell reinsertion into the basal region. Daughters from the same mother are paired. $n = 31$ untreated and 23 STC-treated mitotic cells. NS denotes p-value > 0.05, Wilcoxon Paired Signed Ranks Test.

bioRxiv preprint doi: https://doi.org/10.1101/2025.07.30.665610; this version posted July 31, 2025. The copyright holder for this preprint (which was not certified by peer review) is the author/funder, who has granted bioRxiv a license to display the preprint in perpetuity. It is made available under aCC-BY 4.0 International license.

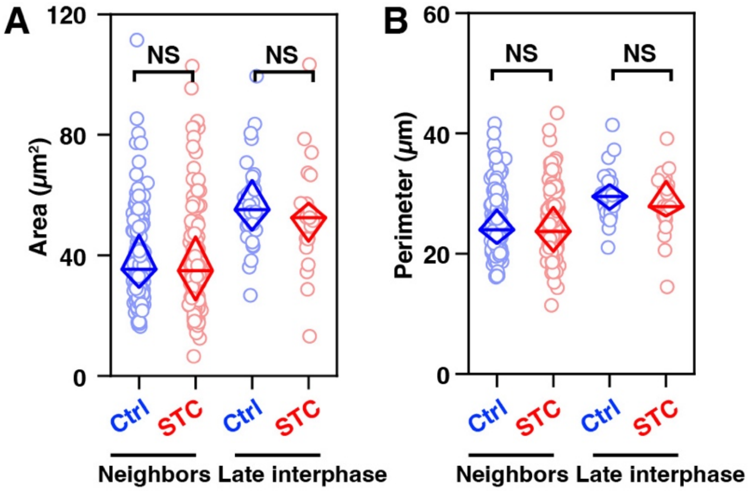

Figure S5. The morphology of STC-treated interphase cells is unaltered
Basal area (A) and perimeter (B) of neighbors of untreated (blue) and STC-treated (red) late interphase cells and their immediate neighbors. *n* = 31 untreated cells and 23 STC-treated prophase cells. NS denotes *p*-value > 0.05, Mann-Whitney Test.
